# Interfacial Electron Engineering for Nitrate-to-Ammonia Electrocatalysis: Mechanistic Insights and Design Strategies

**DOI:** 10.3390/nano16130826

**Published:** 2026-07-05

**Authors:** Xuzhi Liu, Jianqiang Zhu, Zaidong Wang, Han Meng, Yu Ma, Lishi Jiao, Sen Chen, Jian Qi, Huan Wang

**Affiliations:** 1 Hebei Key Laboratory of Flexible Functionals Materials, School of Materials Science and Engineering, Hebei University of Science and Technology, Shijiazhuang 050018, China; drazylm@163.com (X.L.); zjq609205@163.com (J.Z.); wanghbkjstu@163.com (Z.W.); nailuodawang@163.com (H.M.); b431238692026@163.com (Y.M.); neu_cs@163.com (S.C.); 2 State Key Laboratory of Biopharmaceutical Preparation and Delivery, Institute of Process Engineering, Chinese Academy of Sciences, Beijing 100049, China; 3 School of Chemical Engineering, University of Chinese Academy of Sciences, Beijing 100049, China

**Keywords:** nitrate reduction reaction, interfacial electron engineering, built-in electric field, atomic-scale active sites, hydrogen spillover

## Abstract

The electrocatalytic nitrate reduction reaction (NO_3_RR) enables sustainable ammonia synthesis from nitrate waste, yet its complex mechanism and severe competition from the hydrogen evolution reaction (HER) demand precise control over interfacial electronic structures. This review provides a mechanistic overview of interfacial electron engineering for NO_3_RR via charge transfer, d-band center modulation, and d-p orbital coupling. We propose a reverse-engineering framework that starts from the three kinetic bottlenecks of NO_3_RR (nitrate activation, *H supply, and intermediate poisoning) and back-extracts the required electronic effects (charge transfer, d-band shift, and d-p orbital coupling). From this perspective, we cover the construction of built-in electric fields (BIEFs) in heterojunctions, engineering atomic-scale active sites (e.g., single-atom and dual-atom catalysts), and exploiting hydrogen spillover and reverse spillover for cross-spatial proton delivery. Given that rational interfaces dynamically evolve under operating conditions, we highlight that in situ/operando characterization captures the dynamic restructuring of valence states, coordination environments, and morphologies, establishing clear structure–electron–activity relationships. Finally, we discuss key challenges and outline future directions, including machine learning-accelerated screening, dynamic interface regulation, and synergistic integration of multiple electronic effects. This review offers a comprehensive framework for interfacial electron engineering, guiding rational design of next-generation NO_3_RR electrocatalysts.

## 1. Introduction

**Figure 1:**
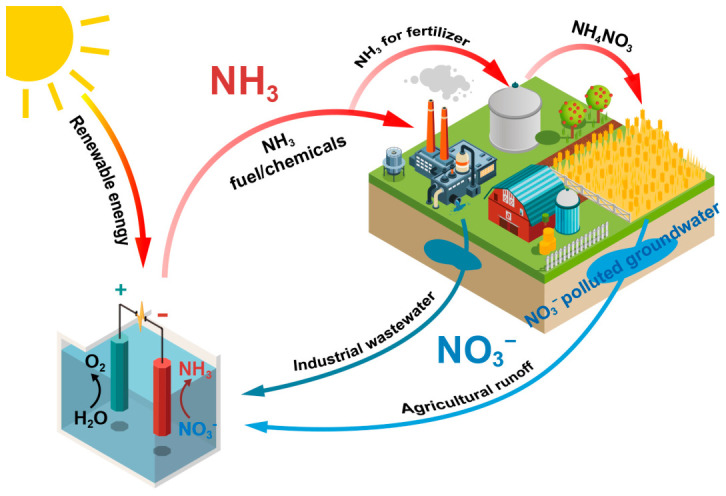
Schematic illustration of sustainable ammonia synthesis from nitrate wastewater via NO_3_RR powered by renewable energy.

Excessive accumulation of nitrate in industrial wastewater and agricultural runoff has become a pressing environmental concern, threatening aquatic ecosystems and human health [1,2,3]. Meanwhile, the conventional Haber–Bosch process for ammonia synthesis, while sustaining global food production, consumes massive fossil energy and emits large quantities of CO_2_ [4,5,6,7]. In this context, the electrocatalytic nitrate reduction reaction (NO_3_RR) has emerged as a win–win strategy addressing both environmental pollution and sustainable energy challenges by converting waste nitrate into high-value ammonia under ambient conditions (Figure 1) [8,9,10]. Unlike the inert N≡N triple bond in N_2_, the relatively low bond energy of N=O bonds in nitrate allows easier activation and reduction, making NO_3_RR a more promising alternative to the nitrogen reduction reaction (NRR) for decentralized ammonia production [11,12,13,14,15].

Nevertheless, NO_3_RR is a complex eight-electron, nine-proton transfer process involving multiple nitrogen-containing intermediates, including NO_2_^−^, NO, and NH_2_OH [16,17,18]. The reaction pathway branches critically at intermediates such as *NO, where undesired by-products (e.g., N_2_) or competing HER can severely lower Faradaic efficiency toward NH_3_ [19,20,21]. Moreover, the thermodynamics of NO_3_RR are strongly influenced by operating conditions, particularly pH and nitrate concentration [22,23,24,25,26]. Under standard conditions, the potential required for NH_3_ production is very close to that of HER, leading to inevitable kinetic competition [27,28]. Therefore, achieving high activity and selectivity requires a fundamental understanding of promoting nitrate activation, optimizing *H supply for hydrogenation, and suppressing parasitic HER simultaneously.

**Figure 2:**
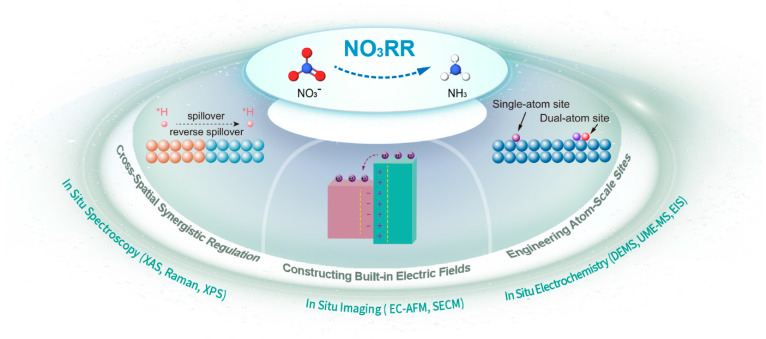
Schematic illustration of key interfacial electron engineering strategies for NO_3_RR.

In recent years, tremendous efforts have been devoted to developing advanced electrocatalysts for NO_3_RR, ranging from noble metals, non-noble metals, and alloys to single-atom catalysts and nonmetallic materials [5,29,30,31]. However, most existing reviews focus on summarizing catalytic materials and performance metrics, often lacking systematic analysis of the underlying electronic principles governing catalyst behavior [4,32,33,34]. Furthermore, while these three core electronic effects, namely interfacial charge redistribution, d-band center modulation, and d-p orbital coupling, have recently gained increasing attention in the context of NO_3_RR as highlighted by several emerging reviews and research articles, a systematic and mechanistic understanding of their synergistic governance of the multi-step reaction pathway remains largely fragmented [35,36,37,38]. In particular, the explicit links between each electronic effect and specific kinetic bottlenecks, including nitrate activation, *H supply, and intermediate poisoning, have not been comprehensively elucidated. This mechanistic gap limits the rational design of next-generation catalysts with optimized activity and selectivity. This review aims to fill this gap by providing a systematic, mechanistic overview of interfacial electron engineering for NO_3_RR (Figure 2). We propose a reverse-engineering framework that starts from the three kinetic bottlenecks of NO_3_RR (nitrate activation, *H supply, and intermediate poisoning) and back-extracts the required electronic effects (charge transfer, d-band shift, and d-p orbital coupling). Unlike traditional forward design that predicts performance from material structures, this reverse approach uses performance requirements as the starting point to guide catalyst architecture. We first elucidate the complex reaction network of NO_3_RR and introduce the three fundamental interfacial electronic effects, namely charge transfer, d-band center modulation, and d-p orbital coupling, that dictate adsorption energetics and reaction pathways. These three effects operate synergistically rather than independently, collectively determining the adsorption-energy landscape of all key intermediates. A reverse-engineering perspective is then presented, including constructing built-in electric fields (BIEFs) in heterojunctions to synergistically enhance nitrate adsorption, *H supply, and intermediate tuning; engineering atomic-scale active sites (e.g., single-atom and dual-atom catalysts) to break linear scaling relations; and exploiting hydrogen spillover and reverse spillover for cross-spatial proton delivery. Recognizing that rationally designed interfaces are not static under operating conditions, advanced in situ/operando characterization techniques (spectroscopy, electrochemistry, and imaging) reveal dynamic evolution of interfacial structures and their profound impact on electronic effects and catalytic performance. This dynamic behavior challenges a purely static view of interfacial electron engineering and establishes clear structure, electron, and activity relationships. Finally, remaining challenges are discussed, and future directions are outlined, including machine learning-accelerated screening, dynamic interface regulation, and the synergistic integration of multiple electronic effects within a single hierarchical architecture. A critical comparison of the three major strategies, BIEFs, atomic-scale active sites, and hydrogen spillover engineering, is also provided to guide strategy selection for specific NO_3_RR applications. By integrating mechanistic insights, design strategies, and dynamic characterization, this review provides a comprehensive framework for interfacial electron engineering toward efficient and selective NO_3_RR electrocatalysis, offering valuable guidance for researchers seeking to transform nitrate waste into a sustainable ammonia resource.

## 2. The Role of Interfacial Electronic Effects in NO_3_RR

NO_3_RR is a complex proton-coupled electron transfer (PCET) reaction network involving eight electrons and nine protons [39,40]. A fundamental understanding of this reaction mechanism is essential for rational catalyst design, because both reaction pathway selectivity and product formation are intrinsically governed by the delicate balance between the electronic structure of the catalyst surface and the adsorption energies of key intermediates.

### 2.1. The Complex Reaction Network of NO_3_RR

Conversion from NO_3_^−^ to NH_3_ proceeds through a series of consecutive deoxygenation and hydrogenation steps, generating various nitrogen-containing intermediates along the pathway. The process begins with the adsorption of NO_3_^−^ onto the catalyst surface, followed by sequential reduction to NO_2_^−^ and NO, ultimately yielding NH_3_ that desorbs from the surface [32,41]. The overall reaction equation and corresponding potential versus the standard hydrogen electrode (SHE) are as follows (Equation (1)) [42,43]:

(1) $${\mathrm{NO}}_{3}^{-}+9H^{+}+8e^{-}\to {\mathrm{NH}}_{3}+3H_{2}O{\;E}^{\circ }=0.69\;V\;\mathrm{vs}.\;\mathrm{SHE}$$

The reaction pathway branches at key intermediates, particularly *NO. *NO can undergo further protonation to form *NOH (leads to NH_3_) or generate *HNO (leading to other by-products). Competition between these pathways, together with unavoidable HER, collectively dictates the ultimate selectivity toward ammonia (Equations (2) and (3)) [8,31,32]. The thermodynamics of NO_3_RR are strongly influenced by operating conditions, particularly pH. Under standard conditions, the potential required for NH_3_ production via NO_3_RR is very close to that of the competing HER, leading to inevitable kinetic competition that directly impacts Faradaic efficiency for ammonia. Therefore, suppressing HER kinetics while supplying sufficient active hydrogen (*H) for hydrogenation steps is key to achieving high NH_3_ selectivity.

(2) $${2\mathrm{NO}}_{3}^{-}+12H^{+}+10e^{-}\to N_{2}+6H_{2}O{\;E}^{\circ }=1.17\;V\;\mathrm{vs}.\;\mathrm{SHE}$$

(3) $$2H^{+}+2e^{-}\to H_{2}{\;E}^{\circ }=0\;V\;\mathrm{vs}.\;\mathrm{SHE}$$

### 2.2. The Interfacial Electronic Effects

Interfacial electronic effects govern NO_3_RR by tuning the adsorption energetics of reactants, intermediates, and final products. These effects mainly involve three fundamental mechanisms: charge transfer, d-band center modulation, and d-p orbital coupling [35,36,37]. Each mechanism plays a distinctive role during the reaction, and they synergistically govern the adsorption–desorption behaviors and electron transfer kinetics of elementary steps.

Charge transfer at catalyst interfaces originates from differences in work function or local electronegativity between dissimilar materials, driving spontaneous electron redistribution and forming electron-rich and electron-deficient regions [44,45]. This charge polarization is critical for NO_3_RR. Electron-deficient sites enhance NO_3_^−^ adsorption through electrostatic interactions, distorting its symmetric structure and elongating N–O bonds, thereby lowering the activation energy barrier for the initial deoxygenation step [45]. Meanwhile, electron-rich sites play a dual role. On one hand, they accelerate water dissociation (a PCET process) to provide a continuous *H supply for subsequent hydrogenation reactions [46]. On the other hand, local electron density modulated by electron-rich regions also governs *H adsorption strength, determining whether *H preferentially participates in targeted hydrogenation or recombines to evolve hydrogen gas. Notably, the spatial distribution and migration behavior of *H on the catalyst surface profoundly influence multi-step hydrogenation efficiency [46]. If *H accumulates locally only at electron-rich sites while NO_x_^−^ adsorption sites lack *H supply, hydrogenation steps become hindered. Conversely, excessive *H coverage on NO_x_^−^ adsorption sites triggers competing HER. Therefore, guiding directional transport of *H through interfacial charge engineering emerges as a key dimension for optimizing the NO_3_RR pathway.

The d-band center, defined as the average energy of metal d-states relative to the Fermi level, serves as a fundamental descriptor for the adsorption strength of adsorbates on transition metal surfaces [35]. According to d-band theory, an upward shift in the d-band center toward the Fermi level strengthens interaction between metal d-orbitals and adsorbate orbitals, thereby enhancing adsorption. Conversely, a downward shift weakens adsorption. This relationship provides a powerful means to optimize adsorption energies of key intermediates within an ideal window following the Sabatier principle [35,46]. In this way, adsorption is neither too strong, which would poison active sites, nor too weak, which would suppress activation. For NO_3_RR, the d-band center must balance competing demands of different reaction steps. An upward shift enhances adsorption of nitrogen-containing intermediates such as NO_3_^−^ and *NO, promoting their activation and subsequent conversion [47]. In contrast, a moderate downward shift weakens binding of strongly adsorbed species like *NO, preventing site poisoning and facilitating product desorption. Furthermore, the d-band center critically influences *H adsorption energy, directly determining hydrogenation step efficiency and HER competition [46]. The *H adsorption energy must be maintained within an optimal range to ensure sufficient *H supply for hydrogenation steps, while avoiding active site occupation from overly strong *H adsorption or insufficient *H generation from overly weak *H adsorption.

The d-p orbital coupling between catalyst active sites and molecular orbitals of adsorbates provides a direct pathway for activating chemical bonds [35,48]. When d-orbitals of transition metals hybridize with antibonding orbitals of N–O bonds, electrons are injected into the antibonding states, thereby weakening the N–O bonds and lowering their dissociation energy barriers [47,49]. This coupling effect is particularly critical for the initial NO_3_^−^ activation step, as the relatively stable N–O bonds must be cleaved to initiate the subsequent reduction cascade. The strength and efficiency of d-p orbital coupling depend on two key factors, energy alignment and symmetry matching [35,50]. The relative energy difference between the metal d-band center and frontier orbitals of the adsorbate determines the filling degree of bonding and antibonding orbitals formed upon coupling. Orbital symmetry, in turn, determines coupling effectiveness, since only orbitals with matched symmetry can overlap effectively. This implies that the catalyst coordination environment not only modulates the energy position of the d-band center but also determines the splitting pattern and the spatial orientation of d-orbitals, thereby influencing their overlap integral with N–O antibonding orbitals [47,51].

These three electronic effects, charge transfer, d-band center modulation, and d-p orbital coupling, do not operate in isolation. Rather, they collectively determine the adsorption-energy landscape of all key intermediates (NO_3_^−^, *NO, *H, and *NH_2_OH) and govern the competition between targeted hydrogenation and parasitic HER. The rational design of NO_3_RR electrocatalysts therefore requires engineering these effects synergistically rather than independently. In the following section, we examine the potential for realizing such synergistic engineering through specific catalyst architectures, including heterojunctions with built-in electric fields, atomically dispersed active sites, and hydrogen spillover interfaces.

## 3. Reverse-Engineering Interfacial Electronic Effects for Catalyst Design

The three electronic effects discussed above, namely charge transfer, d-band center modulation, and d-p orbital coupling, are closely interrelated. The central challenge is to synergistically integrate these effects into a single catalyst system to tackle the three kinetic bottlenecks of NO_3_RR: nitrate activation, *H supply, and intermediate poisoning. In this section, we review three representative design strategies developed to address this challenge: constructing built-in electric fields in heterojunctions, engineering atomic-scale active sites such as single-atom and dual-atom catalysts, and exploiting hydrogen spillover, including reverse spillover, for cross-spatial proton delivery.

To provide a performance-oriented overview of these strategies, Table 1 summarizes the catalytic performance of representative NO_3_RR electrocatalysts reported in recent years, including electrolyte conditions, NH_3_ yield rate, and Faradaic efficiency. The following subsections elaborate on the design principles, mechanistic origins, and structure–electron–activity relationships underlying these performance data.

### 3.1. Constructing Built-In Electric Fields

NO_3_RR involves a complex eight-electron and nine-proton transfer process, whose kinetic bottleneck is mainly determined by nitrate adsorption and activation together with efficient *H supply. Furthermore, adsorption strength of key intermediates such as *NO directly influences the reaction pathway selectivity. Achieving simultaneous optimization of these processes within a single catalyst system is crucial for enhancing NO_3_RR performance. In recent years, constructing built-in electric fields (BIEFs) has emerged as an effective strategy to address this challenge [52,53,68]. A BIEF refers to the space-charge region formed at the interface when two materials with distinct work functions (Φ) come into intimate contact, driving spontaneous electron transfer from the material with lower work function to that with higher work function until Fermi-level equilibration [69,70]. The presence of BIEFs induces interfacial charge redistribution, forming complementary electron-rich and electron-deficient regions, thereby providing an ideal platform for achieving multidimensional synergy encompassing nitrate adsorption, *H supply, and even intermediate adsorption optimization.

To validate this concept, several heterojunction catalysts have been rationally designed to harness BIEFs for simultaneous optimization of nitrate adsorption, *H supply, and intermediate binding in NO_3_RR. Chen et al. successfully fabricated a CuO/NiO heterojunction, systematically elucidated the BIEF formation mechanism, and demonstrated its pivotal role in dual-functional enhancement of low-concentration NO_3_RR [52]. Ultraviolet photoelectron spectroscopy (UPS) measurements revealed a 0.21 eV work function difference between CuO and NiO, driving spontaneous electron transfer from NiO to CuO across the heterointerface. This interfacial electron transfer was further corroborated by X-ray photoelectron spectroscopy (XPS) analysis. By combining experimentally determined work functions with literature bandgap values, a detailed energy band diagram was constructed, unambiguously confirming establishment of BIEFs at the heterojunction interface. Density functional theory (DFT) calculations demonstrated that adsorption energies of NO_3_^−^ and NO_2_^−^ on the CuO/NiO interface (−1.90 eV and −1.29 eV, respectively) are significantly higher than those on pristine CuO and NiO. This indicates that BIEF-induced charge polarization markedly enhances enrichment and mass transfer of low-concentration reactants at the interface. Electron spin resonance (ESR) spectroscopy confirmed participation of *H in the reduction process. Furthermore, DFT calculations revealed that CuO/NiO exhibits moderate *H adsorption energy (−0.64 eV), effectively balancing the trade-off between insufficient *H supply on pristine CuO (−0.71 eV) and excessive *H adsorption on pristine NiO (−0.31 eV) that would otherwise trigger competitive HER. Benefiting from BIEF-driven synergistic effects, the CuO/NiO heterojunction achieved exceptional performance in 100 mg L^−1^ NO_3_^−^ solution, attaining 100% nitrate conversion, 100% NH_4_^+^ selectivity, and 61.0% Faradaic efficiency [52]. This work provides comprehensive experimental and theoretical evidence clarifying how a BIEF enhances low-concentration NO_3_RR, offering valuable insights for rational design of high-performance electrocatalysts. Subsequently, Zhao et al. further investigated the impact of BIEF on *H supply kinetics [53]. By constructing a Co_3_O_4_/CeO_2_ heterojunction, they revealed that a BIEF oriented from CeO_2_ to Co_3_O_4_, together with nitrogen-doped carbon nanotubes (NCNTs), synergistically optimizes the *H supply process. More critically, under BIEF regulation, CeO_2_ serves as an efficient center for water adsorption and dissociation, continuously supplying *H for nitrate hydrogenation steps on adjacent Co_3_O_4_ sites, while its own HER activity is effectively suppressed. ESR experiments further confirmed that the Co_3_O_4_/CeO_2_ heterojunction enables more efficient *H generation and rapid consumption in hydrogenation reactions. This site-specific relay mechanism endows Co_3_O_4_/CeO_2_@NCNTs with an exceptional NH_3_ yield rate of 14.85 mg h^−1^ cm^−2^ and Faradaic efficiency exceeding 97.1% in 1.0 M NO_3_^−^, along with remarkable stability over 120 h [53]. This work extends understanding of BIEFs from reactant adsorption to *H supply kinetics, offering a precise interpretation of how a BIEF overcomes the NO_3_RR kinetic bottleneck by optimizing *H availability.

**Figure 3:**
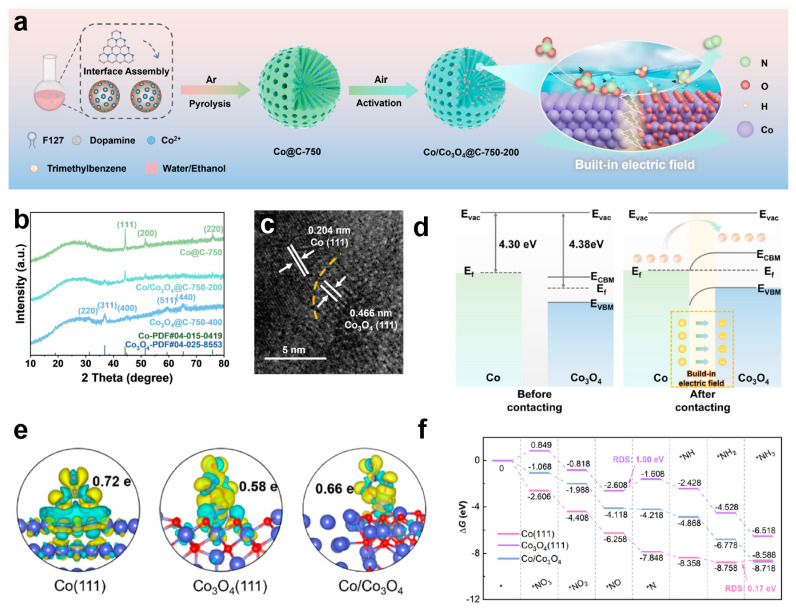
Synthesis and characterization of Co/Co_3_O_4_@C-750-200. (**a**) Schematic illustration for the synthesis of Co/Co_3_O_4_@C-750-200. (**b**) XRD patterns. (**c**) HAADF−STEM image of Co/Co_3_O_4_@C-750-200. (**d**) Schematic illustration of the Co and Co_3_O_4_ before and after contact. (**e**) Bader charge transfer and charge density difference between the three systems and adsorbed NO_3_^−^. (**f**) Gibbs free energy diagram for NO_3_RR via the most favorable pathway on Co(111), Co_3_O_4_(111), and the Co/Co_3_O_4_ heterostructure interface. Reprinted with permission from ref. [68], Copyright 2025, American Chemical Society.

In addition, the adsorption behavior of key intermediates plays a decisive role in determining NO_3_RR reaction pathway selectivity. In this context, Fu et al. constructed a CoO/Cu heterojunction to systematically investigate how a BIEF optimizes reaction selectivity by modulating the adsorption strength of the critical *NO intermediate [54]. Experimental analyses and DFT calculations revealed that a 1.32 eV band offset between the valence band maximum of CoO and the Fermi level of Cu drives electron transfer from Cu to CoO, establishing a BIEF directed from Cu toward CoO. This charge redistribution endows interfacial Cu atoms with a slight positive charge of +0.01 eV and CoO with a slight negative charge of −0.007 eV. Such a unique charge configuration enables a dual optimization effect. Specifically, positively charged Cu sites enhance NO_3_^−^ adsorption through electrostatic interactions, while negatively charged CoO sites donate electrons into antibonding orbitals of *NO, significantly weakening its adsorption strength from −3.89 eV to −2.19 eV. Given that weakened *NO adsorption benefits NH_3_ selectivity, this BIEF-mediated intermediate adsorption modulation effectively optimizes the reaction pathway toward desirable products. Benefiting from synergistic optimization of both reactant and intermediate adsorption, the CoO/Cu foam electrode delivers an impressive NO_3_^−^ removal rate of 87.6%, along with 97.3% NH_3_ selectivity and 96.7% Faradaic efficiency. Recent studies have progressively uncovered diverse roles of BIEFs in reactant adsorption, proton supply, and intermediate modulation. Taking this further, Xie et al. successfully integrated these functions into a mesoporous carbon-supported Co/Co_3_O_4_ heterojunction catalyst through a facile emulsion self-assembly strategy (Figure 3a–c) [68]. DFT calculations visually confirmed the spatial synergistic mechanism underlying this system. Results show that the higher Fermi level of Co drives spontaneous electron transfer from Co to Co_3_O_4_, creating a BIEF directed from Co toward Co_3_O_4_, as schematically illustrated in Figure 3d. This charge redistribution is further evidenced by the charge density difference analysis (Figure 3e), which reveals electron accumulation and depletion at the heterointerface. The Gibbs free energy diagram (Figure 3f) further validates that the heterojunction significantly reduces the reaction barriers along the optimal NO_3_RR pathway, thereby enabling superior catalytic performance. Based on this synergistic mechanism, the Co/Co_3_O_4_ catalyst delivers a 90.77% NO_3_^−^ removal rate and up to 99% N_2_ selectivity under neutral conditions with low-concentration nitrate.

**Figure 4:**
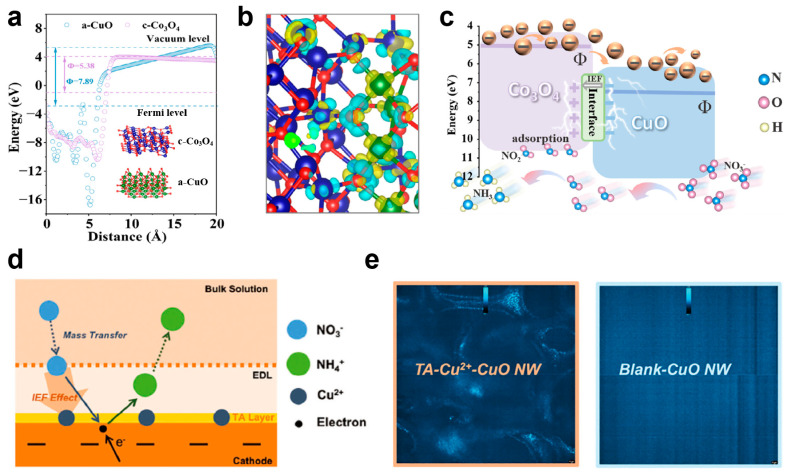
(**a**) Work functions obtained from DFT calculations. (**b**) Differential charge density of c-Co_3_O_4_/a-CuO (yellow and blue regions representing charge accumulation and depletion, respectively). (**c**) Mechanism diagram of NO_3_RR performed by c-Co_3_O_4_/a-CuO. Reprinted with permission from ref. [55], Copyright 2025, Wiley-VCH GmbH. Mechanism investigation of IEF effect on TA-Cu^2+^-CuO NW/Cu foam. (**d**) Schematic of proposed IEF mechanism for nitrate ions. (**e**) Fluorescence microscopy images of TA-Cu^2+^-CuO NW/Cu foam and Blank-CuO NW/Cu foam. Reprinted with permission from ref. [59], Copyright 2024, The Author(s), Advanced Science published by Wiley-VCH GmbH.

Following the BIEF design concept, researchers have developed various heterostructure construction strategies, further expanding its application boundaries in NO_3_RR. In terms of interfacial crystal phase engineering, crystalline-amorphous heterojunctions exhibit unique advantages in electronic modulation. Chen et al. constructed a c-Co_3_O_4_/a-CuO heterojunction, where the flexible coordination environment of amorphous CuO induces stronger charge localization compared to conventional crystal–crystal interfaces (Figure 4a–c) [55]. Meanwhile, Zhang et al. introduced oxygen vacancies into Bi_2_S_3_-Bi_2_O_3_ heterojunctions to further enhance BIEFs through defect engineering [56]. He et al. employed an electrochemical reconstruction strategy to construct a Co(OH)_2_/CoO heterojunction within a single-metal Co system, demonstrating that a BIEF is not exclusive to multi-metal systems [57]. Regarding material system expansion, the BIEF strategy has been successfully extended to various non-oxide systems. The Cu_x_S-Co_0.5_ sulfide heterojunction synthesized by Zhang et al. and the FeP_4_/Ni_2_P phosphide heterojunction reported by Lv et al. both confirm that interfacial electronic coupling promotes *H generation [58,71]. Additionally, Gao et al. utilized the Schottky junction between Ni and N-doped carbon to induce a surface electric field, providing a new approach for NO_2_^−^ reduction [72]. In terms of interfacial construction methods, researchers have also explored novel approaches beyond traditional semiconductor heterojunctions. Zhang et al. developed an organic–metal complex interfacial regulation strategy by constructing a TA-Cu^2+^ complex layer on the CuO surface, forming a stable positive charge enrichment region that concentrates NO_3_^−^ through the interfacial electric field (Figure 4d,e) [59].

While the aforementioned BIEF strategies work under steady-state potentials, a recent study by Wang et al. combined built-in electric fields with pulsed electroreduction to realize efficient conversion of low-concentration nitrate [73]. Employing a biochar-supported Co_3_O_4_/Fe_3_O_4_ heterojunction (Co_2_Fe_1_/C) with intrinsic BIEFs, they adopted alternating cathodic and anodic pulses to replenish interfacial nitrate ions and suppress the formation of depletion layers during cathodic reduction. In a neutral electrolyte containing 0.01 M NO_3_^−^, the pulsed mode increased the ammonia Faradaic efficiency from 55.6% to 73.4% and achieved an NH_3_-N selectivity of 93.4%, alongside a 21% reduction in energy consumption for ammonia synthesis. This study demonstrates that dynamic potential modulation can further enhance the performance of BIEFs, an approach that has been largely unexplored in traditional heterojunction catalysts for NO_3_RR. Integrating BIEFs with non-steady-state electrolysis in future research will help optimize the trade-off among nitrate activation, *H supply and HER inhibition.

These cases discussed above collectively demonstrate that constructing a BIEF provides an effective strategy to simultaneously address multiple kinetic bottlenecks in NO_3_RR, including nitrate activation, *H supply, and the competing hydrogen evolution reaction. A key advantage of a BIEF lies in its ability to induce complementary charge distributions across heterointerfaces, thereby allowing adjacent domains to assume distinct catalytic roles. For example, electron-deficient regions enhance NO_3_^−^ adsorption, whereas electron-rich zones promote water dissociation. The scope of this approach has since broadened to include crystalline–amorphous junctions, defect-rich interfaces, and even monometallic reconstructed systems. Nevertheless, the evolution of a BIEF under applied potentials remains poorly understood, a question that operando characterizations are only beginning to explore. Equally critical is the long-term stability of these heterointerfaces against delamination or phase segregation. Without such knowledge, translating BIEF-based catalysts from idealized laboratory conditions to practical wastewater electrolysis will remain a significant challenge.

### 3.2. Engineering Atomic-Scale Sites

As interface engineering in heterostructures extends electronic modulation to the nanoscale, achieving quantum-level control over interfacial electronic states at the atomic scale has emerged as a key scientific challenge in the field. Single-atom catalysts provide an ideal platform to address this challenge [60,74,75,76]. Unlike two-dimensional continuous interfaces formed by work function differences between two materials, the interface in single-atom catalysts is confined to a zero-dimensional local coordination environment, specifically the chemical bonding region between the central metal atom and its surrounding coordination atoms [77,78]. The electronic structure of this atomic-scale interface is determined by the symmetry, electronegativity, and covalency of the coordination field, providing exceptional tunability for precisely controlling the eight-electron transfer in NO_3_RR.

#### 3.2.1. Single-Atom Site

In single-atom catalysts, reactant adsorption and activation energy barriers are directly correlated with the d-band center position of the central metal. By tailoring the central metal type or its coordination environment, continuous and precise modulation of the d-band center can be achieved. This enables optimization of key intermediate adsorption energies to an ideal range in accordance with the Sabatier principle. Wang et al. systematically evaluated the NO_3_RR performance of 23 transition metal single atoms with TM–N_4_ configuration through high-throughput DFT calculations and constructed a volcano plot using ΔG_*NO3_ as the descriptor [74]. Their findings reveal that, under the identical N_4_ coordination environment, simply changing the central metal atom can significantly alter interfacial electronic structure. Specifically, while both Fe–N_4_ and Os–N_4_ lie near the volcano summit, the Os single-atom catalyst exhibits a superior limiting potential (−0.42 V vs. −0.53 V). Electronic structure analysis further uncovers that this difference originates from stronger charge transfer (1.43 e^−^) and more pronounced d-p orbital coupling between the Os atom and NO_3_^−^. This work directly demonstrates that tuning the central metal at the atomic scale can reshape interfacial electronic structure, thereby governing the rate-determining step of the reaction.

**Figure 5:**
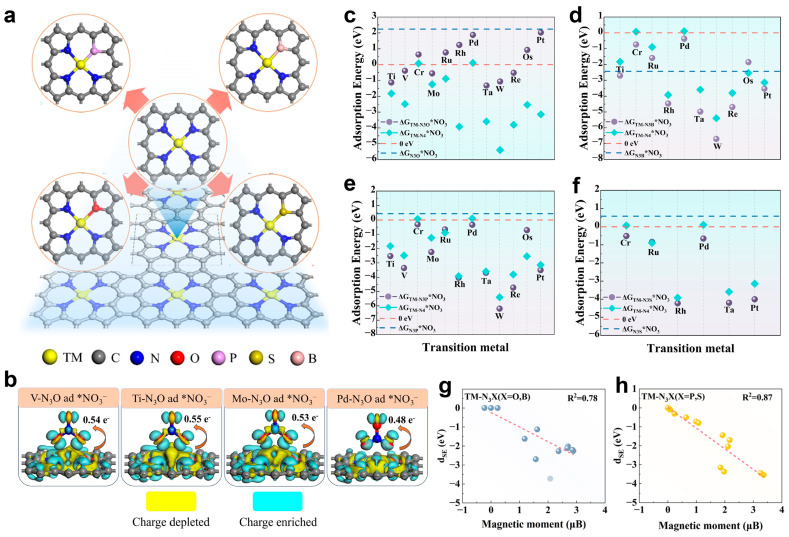
(**a**) Four TM–N_3_X configurations (TM = Ti, V, Cr, Nb, Mo, Ru, Rh, Pd, Ta, W, Re, Os, Pt) derived from TM–N_4_, including TM–N_3_O, TM–N_3_P, TM–N_3_S and TM–N_3_B. (**b**) Charge density differences of NO_3_^−^ adsorbed on V–N_3_O, Ti–N_3_O, Mo–N_3_O and Pd–N_3_O. Adsorption energy of 40 TM–N_3_X catalysts for NO_3_^−^ adsorption in corresponding modes. (**c**) TM–N_3_O. (**d**) TM–N_3_B. (**e**) TM–N_3_P. (**f**) TM–N_3_S. The figure shows the NO_3_^−^ adsorption energy of the TM–N_4_ structure of the corresponding metal. (**g**,**h**) Relationship between the multiorbital splitting energy (d_SE_) and the transition metal magnetic moment. Reprinted with permission from ref. [47], Copyright 2025, Royal Society of Chemistry.

Tailoring the coordination environment involves more than just changing the central metal. Wu et al. systematically investigated 52 TM–N_3_X (X = O, P, S, B) configurations using DFT calculations, revealing the profound influence of heteroatom-induced symmetry breaking on the electronic structure (Figure 5a) [47]. Charge density difference calculations (Figure 5b) intuitively exhibit the charge depletion and accumulation around metal active sites, directly evidencing the electronic structure rearrangement induced by symmetry breaking. The study reveals that doping with P, S, or B lowers or preserves the intermediate adsorption energy, thereby favoring reactant activation, whereas O doping increases the adsorption energy, resulting in weakened adsorption (Figure 5c–f). More critically, symmetry breaking induced by heteroatom doping significantly affects the multiorbital splitting energy (*d*_SE_) and magnetic moment of the central metal (Figure 5g,h), thereby modulating bonding and antibonding states of intermediates. This discovery expands the theoretical understanding of coordination field engineering in single-atom catalysts to the orbital energy level, offering a new electronic structure perspective on the role of different coordination environments in governing catalytic activity.

The type of nitrogen within the coordination environment is also critical. Liu et al. fabricated a pyridinic-N-rich Cu single-atom catalyst (PR-CuNC) and revealed the finely tuned role of nitrogen coordination structures on electronic states [60]. Compared to CuNC with lower pyridinic-N content, PR-CuNC exhibited significantly enhanced NO_3_RR performance, achieving a 94.61% Faradaic efficiency and a 3.74 mg h^−1^ cm^−2^ NH_3_ yield rate. Theoretical calculations demonstrate that different nitrogen coordination types (pyridinic-N and pyrrolic-N) distinctly influence the electronic structure of the Cu–N_4_ site. Specifically, pyridinic-N coordination induces stronger coupling between Cu d-orbitals and N p-orbitals, along with a more concentrated charge distribution. This leads to a lower energy barrier of 0.35 eV for *NO hydrogenation to *NHO, compared to 0.52 eV for pyrrolic-N coordination, thereby greatly enhancing the reactivity of this step.

In addition, diverse single-atom structures have been developed for NO_3_RR. These include TM–N_4_ sites with different central metals (e.g., Fe, Co, Ni, Cu, Ru, Os, Ti, Zr), TM–N_3_X sites with heteroatom doping (X = O, P, S, B), and carbon-supported single atoms without nitrogen coordination (e.g., B-doped diamond, B-doped carbon dots) [79,80,81]. The coordination environment has been further refined by tuning the nitrogen type (pyridinic vs. pyrrolic N) [26,82]. In terms of support materials, beyond N-doped carbon, single atoms have also been anchored on metal oxides, MOFs, and MXenes [83,84,85]. Thus, coordination field engineering can precisely tailor the interfacial electronic structure of single-atom catalysts for NO_3_RR. Tuning the central metal, coordination environment and heteroatom doping allows continuous modulation of the d-band center and d-p orbital coupling, thus optimizing the adsorption of key *NO_3_ and *NO intermediates. Nevertheless, several challenges still exist. Single-atom sites are prone to structural degradation and aggregation under reaction potentials, while low site density also limits the catalytic current density and practical application. In addition, precise regulation of pyridinic and pyrrolic nitrogen ligands remains difficult in synthesis. Future research should focus on monitoring catalyst structural evolution under real reaction conditions, developing scalable synthetic methods to achieve high single-atom loading without sacrificing atomic dispersion, and designing suitable conductive supports to boost electron transfer efficiency.

The central insight from single-atom catalyst studies is that coordination field engineering offers precise, site-specific control over interfacial electronic structure. By adjusting the central metal, coordination environment, or heteroatom doping, one can continuously tune the d-band center and d–p orbital coupling to optimize the adsorption of *NO_3_ and *NO intermediates. Despite these advances, a common challenge across all single-atom systems is the structural degradation and aggregation of isolated sites under reaction potentials, which directly undermines the long-term stability of the tailored electronic effects. Furthermore, the low site density of single-atom catalysts limits the achievable current density, hindering their practical application. Future efforts should focus on monitoring the structural evolution of single-atom sites under real reaction conditions using operando techniques, developing scalable synthesis methods to achieve high metal loading without sacrificing atomic dispersion, and designing conductive supports to boost electron transfer efficiency.

#### 3.2.2. Dual-Atom Site

Beyond single-atom sites, diverse dual-atom configurations have also been developed for NO_3_RR. These include heteronuclear pairs such as Cu–Fe, Fe–Mn, Ru–Cu, Co–Fe, Ni–Fe, and Pd–Cu, as well as homonuclear dual-atom sites (e.g., Cu–Cu) [61,62,63,86,87,88,89]. The interatomic distance between the two metal sites, typically in the range of 2.5–3.5 Å, is a critical parameter that determines the extent of electronic coupling and the feasibility of tandem or asynchronous mechanisms [90]. Asymmetric coordination, where the two metal centers adopt different ligand environments (e.g., N_3_–Fe–Mn–N_2_S, RuN_2_–CuN_3_), has emerged as a particularly effective strategy to further break linear scaling relations [62,63]. A variety of support materials have been employed for anchoring dual-atom sites, including N-doped carbon, graphene aerogels, and metal–organic frameworks [61,63,91,92,93]. Despite the diversity of dual-atom configurations, several common design principles can be summarized. The synergistic interaction between adjacent metal sites enables functional differentiation, where one site is responsible for nitrate activation and the other for hydrogenation or *H supply. The interatomic distance and coordination asymmetry are key parameters governing the degree of electronic coupling and the efficiency of the tandem mechanism.

The Cu–Fe dual-atom catalyst (Cu–Fe DAC/NC) reported by Maeng et al. supports this concept [61]. Both Cu and Fe are atomically dispersed and distributed adjacently on the nitrogen-doped carbon support. Electronic interaction between them forms a structurally coupled dual-atom site with an interatomic distance of approximately 2.6 Å. Electrochemical tests demonstrate that Cu–Fe DAC/NC achieves 94.3% Faradaic efficiency and a 6.0 mg cm^−2^ h^−1^ NH_3_ yield rate at −0.53 V vs. RHE, significantly outperforming corresponding single-atom catalysts Cu SAC and Fe SAC [61]. Mechanistic studies reveal an elegant tandem mechanism in which the Cu site activates NO_3_^−^, while the Fe site hydrogenates NO_2_^−^. This cooperative dual-atom mode breaks the activity limitations imposed by linear scaling relations on single sites, offering important guidance for designing dual-atom catalysts for multistep reactions.

**Figure 6:**
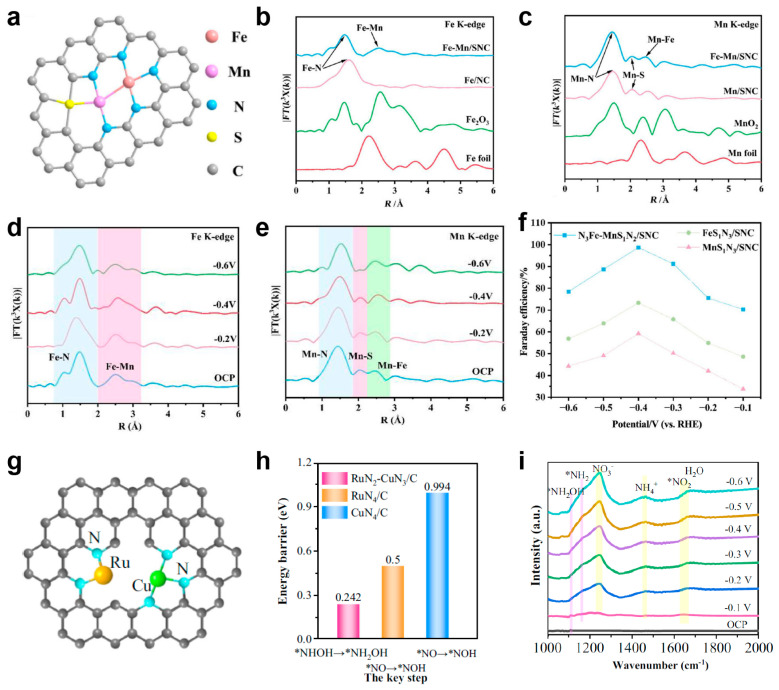
(**a**) Atomic structure model of Fe-Mn/SNC. The FT-EXAFS spectra of Fe-Mn/SNC and the references at (**b**) Fe K-edge and (**c**) Mn K-edge. In situ (**d**) Fe K-edge and (**e**) Mn K-edge FT-EXAFS at open circuit, −0.2 V, −0.4 V and −0.6 V vs. RHE. (**f**) The Faradaic efficiencies of NH_3_ production at varied potentials. Reprinted with permission from ref. [62], Copyright 2026, SIOC, CAS, Shanghai, and WILEY-VCH GmbH. (**g**) The proposed atomic structure model of RuCuDAs/NGA (yellow, Ru; green, Cu; cyan, N; gray, C). (**h**) The energy barriers of the key steps on RuN_4_/C, CuN_4_/C, and RuN_2_CuN_3_/C. (**i**) Potential-dependent in situ ATR-SEIRAS spectra during the NO_3_RR process. Reprinted with permission from ref. [63], Copyright 2025, published under a CC BY 4.0 license.

Extending dual-atom catalyst design strategy from symmetric to asymmetric coordination, Wang et al. constructed a Fe–Mn heteronuclear dual-atom site (Fe–Mn/SNC) featuring a unique lateral asymmetric coordination structure [62]. Its specific configuration is N_3_–Fe_1_–Mn_1_–N_2_S, with Fe coordinated by three N atoms and Mn by two N atoms and one S atom (Figure 6a). This asymmetric coordination enables modulation through both coordinating atom type (N-only vs. N/S coexistence) and coordination environment heterogeneity. In situ X-ray absorption spectroscopy (XAS) and DFT calculations revealed a strong electronic coupling effect between the Fe and Mn sites, as well as a synergistic modulation of the electronic structure by S and N atoms (Figure 6b,c). Specifically, the S atom regulates Mn electron density through the Mn–S bond, while N atoms stabilize the Fe coordination environment; this synergistic effect optimizes intermediate adsorption strength (Figure 6d,e). This asymmetric coordination design not only balances key intermediate adsorption energies but also enhances catalyst structural stability, ensuring an intact coordination environment during the reaction. As a result, Fe–Mn/SNC achieves 98.7% Faradaic efficiency at a low potential of −0.4 V vs. RHE, demonstrating excellent NO_3_RR selectivity (Figure 6f).

In addition, coupling noble metals (e.g., Ru, Pd, Rh) with transition metals (e.g., Cu, Co) to construct heteronuclear dual-atom sites offers another effective dimension for precise modulation of interfacial electronic states and reaction pathways [63,88,94,95]. The distinctive electronic structures of noble metals, in synergy with transition metals, can effectively overcome performance limitations typically associated with conventional single active centers. A Ru–Cu dual-atom catalyst (RuCu DAs/NGA) was successfully synthesized and applied for NO_3_RR [63]. Using a pulsed discharge strategy, dual-atom sites with an asymmetric RuN_2_–CuN_3_ coordination structure were constructed on nitrogen-doped graphene aerogel (Figure 6g). This asymmetric coordination induces strong interfacial electronic coupling, enabling Ru and Cu sites to undertake distinct catalytic functions. DFT calculations and in situ spectroscopy reveal that the Ru center optimizes the adsorption energy of nitrogen-containing intermediates such as *NO, reducing the key energy barrier for the *NO → *NOH step to 0.242 eV and significantly facilitating N–O bond cleavage (Figure 6h). Meanwhile, the Cu center contributes to the reaction pathway through electron transfer and synergistic adsorption, co-adsorbing intermediates like NO_3_^−^ and *NO with Ru to create favorable conditions for hydrogenation steps. In situ ATR-SEIRAS in Figure 6i further tracked the complete reaction pathway of NO_3_^−^ → *NO_2_ → *NO → *NH_2_OH → *NH_3_, confirming efficient conversion of intermediates. Benefiting from this bimetallic synergy, RuCu DAs/NGA achieves a high Faradaic efficiency of 95.7% and an ammonia yield of 3.1 mg h^−1^ cm^−2^ at −0.4 V vs. RHE [63].

Dual-atom catalysts stand as an appealing alternative to break the bottlenecks of single-site catalysts, thanks to the synergistic and tandem mechanisms derived from paired metal centers. Tuning the metal pair combination, interatomic distance, and coordination asymmetry allows precise modulation of the d-band center and interfacial charge distribution, thereby optimizing the adsorption energies of multiple intermediates simultaneously. Nevertheless, several challenges remain. The synthesis of well-defined dual-atom sites with precise control over interatomic distance and coordination symmetry is still challenging, and most reported methods suffer from low yield and poor uniformity. Moreover, the dynamic evolution of dual-atom sites under reaction potentials, including potential metal aggregation or site separation, remains poorly understood. Future research should focus on developing more controllable synthetic strategies, utilizing in situ characterization techniques to track the structural evolution of dual-atom sites during NO_3_RR, and exploring the incorporation of dual-atom sites into conductive frameworks to enhance electron transfer.

Dual-atom catalysts distinguish themselves from single-atom systems through the synergistic and tandem mechanisms enabled by paired metal centers. Tuning the metal pair combination, interatomic distance, and coordination asymmetry allows precise modulation of the d-band center and interfacial charge distribution, thereby optimizing the adsorption energies of multiple intermediates simultaneously. A distinctive advantage of dual-atom systems is their ability to decouple the adsorption of different intermediates through functional differentiation of the two metal centers, offering a pathway to overcome linear scaling relations. Nevertheless, several common challenges persist. The synthesis of well-defined dual-atom sites with precise control over interatomic distance and coordination symmetry is still challenging, and most reported methods suffer from low yield and poor uniformity. Moreover, the dynamic evolution of dual-atom sites under reaction potentials, including potential metal aggregation or site separation, remains poorly understood. Future research should focus on developing more controllable synthetic strategies, utilizing in situ characterization to track the structural evolution of dual-atom sites during NO_3_RR, and exploring the incorporation of dual-atom sites into conductive frameworks to enhance electron transfer.

### 3.3. Cross-Spatial Synergistic Regulation

#### 3.3.1. Hydrogen Spillover

As a BIEF redistributes at heterojunction interfaces and single-atom catalysts tailor the d-band center at the coordination field scale, a deeper kinetic challenge for the nine-proton-transfer NO_3_RR process is achieving efficient generation and directional transport of *H [96]. Strategic utilization of the hydrogen spillover effect provides a unique approach for realizing spatial transfer of *H from hydrogen evolution sites to hydrogenation sites [97]. Upon formation of an intimate heterojunction interface between two materials with distinct hydrogen adsorption capabilities, *H can spontaneously migrate from sites with weaker hydrogen adsorption to those with stronger hydrogen adsorption until chemical potential equilibrium is reached [97]. This process enables spatial separation of *H generation sites from consumption sites, effectively suppressing competing HER caused by excessive *H coverage on active sites while ensuring stable *H supply for multi-step hydrogenation reactions [65].

**Figure 7:**
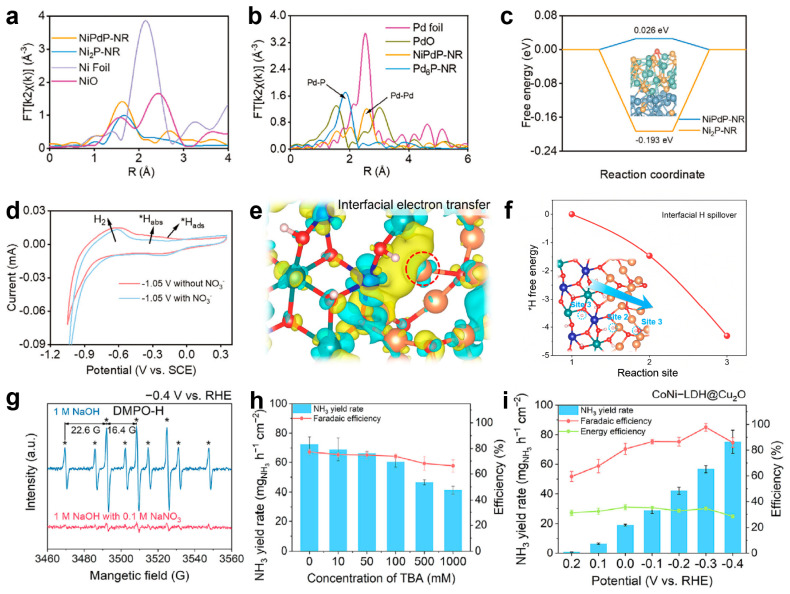
(**a**) Fourier transform of Ni K-edge EXAFS for NiPdP-NR and Ni_2_P-NR. (**b**) Fourier transform of Pd K-edge EXAFS for NiPdP-NR and Ni_2_P-NR. (**c**) DFT-calculated Hydrogen adsorption energies on the Ni_2_P-NR and NiPdP-NR. (**d**) CV curves of the NiPdP-NR. Reprinted with permission from ref. [64], Copyright 2023, WILEY-VCH GmbH. (**e**) Charge density difference in the interface of the CoNi–LDH@Cu_2_O heterostructure, where yellow color represents charge accumulation and blue color represents charge depletion. (**f**) *H free energy at different sites across the interface between CoNi–LDH and Cu_2_O. The inset represents the spontaneous hydrogen spillover process. (bule, Co; green, Ni; orange, Cu; red, O; light pink, H). (**g**) In situ EPR spectra of CoNi–LDH@Cu_2_O at −0.4 V vs. RHE. (**h**) Performance comparison of CoNi–LDH@Cu_2_O. (**i**) NH_3_ yield rate, Faradaic efficiency, and energy efficiency of CoNi–LDH@Cu_2_O at different potentials. Reprinted with permission from ref. [65], Copyright 2025, WILEY-VCH GmbH.

The Ni_2_P/Pd_6_P porous nanorod system constructed by Zhang et al. systematically revealed the contribution of classical hydrogen spillover to NO_3_RR [64]. Incorporation of Pd enabled the catalyst to achieve 92.6% Faradaic efficiency and a 0.908 mmol h^−1^ mg_cat_^−1^ ammonia yield rate at −0.645 V vs. RHE, significantly outperforming pristine Ni_2_P and Pd_6_P. The core mechanism lies in Pd sites serving as *H producers due to superior water dissociation capability, while Ni_2_P sites function as *H consumers responsible for hydrogenating nitrogen-containing intermediates. XPS and X-ray absorption fine structure (XAFS) spectroscopy confirmed strong electronic interaction between Pd and Ni_2_P, which optimized *H adsorption energy on the catalyst surface to 0.026 eV (Figure 7a–c). This value is neither too strong to cause catalyst poisoning nor too weak to hinder reaction participation. Cyclic voltammetry (Figure 7d) measurements further demonstrated that *H generated on Pd sites can be effectively consumed by NO_3_^−^, confirming the bridging role of hydrogen spillover in coupling *H supply with utilization.

Beyond the classical hydrogen spillover mechanism exemplified by the Ni_2_P/Pd_6_P system, recent studies have further explored the coupling of hydrogen spillover with interfacial electron transfer to achieve dual synergistic enhancement of NO_3_RR performance. Zheng et al. further demonstrated dual synergy of hydrogen spillover and interfacial electron transfer by constructing CoNi-layered double hydroxide-modified Cu_2_O nanowires (CoNi-LDH@Cu_2_O) [65]. DFT calculations revealed that the work function difference between CoNi-LDH (Φ = 1.86 eV) and Cu_2_O (Φ = 4.55 eV) drives electron transfer from CoNi-LDH to Cu_2_O. As shown in Figure 7e, charge density difference analysis confirms charge accumulation around the interfacial Cu atoms and charge depletion on the CoNi-LDH side, indicating that the transferred electrons enrich the Cu sites. This increased electron density at the Cu sites facilitates the reduction in adsorbed NO_3_ by weakening the N–O bonds through electron injection into their antibonding orbitals (Figure 7f). More critically, CoNi-LDH serves as a *H producer due to its excellent water dissociation capability. Generated *H then migrates via interfacial hydrogen spillover to adjacent Cu_2_O sites for hydrogenation of nitrogen-containing intermediates. In situ EPR spectroscopy (Figure 7g) confirmed *H generation and consumption, and tert-butanol quenching experiments (Figure 7h) verified that *H is the key active species in NO_3_RR. Benefiting from dual synergy of hydrogen spillover and electron transfer, CoNi-LDH@Cu_2_O achieves 97.8% Faradaic efficiency at −0.3 V vs. RHE and a 75.2 mg h^−1^ cm^−2^ ammonia yield rate at −0.4 V vs. RHE (Figure 7i).

This design principle has also been extended to other material platforms, including phosphide-based heterostructures (e.g., CoP/TiO_2_ [98], NiCoP/C [99]) and metal–carbon hybrids (e.g., Pd/N-doped carbon [100]). In all these systems, a water-dissociation component is paired with a nitrate-hydrogenation component, and the difference in *H adsorption energy is used to guide the design. Nevertheless, several challenges remain. Direct nanoscale observation of *H migration is still lacking, and most evidence is indirect. In addition, nearly all spillover-enhanced catalysts have only been tested in high-concentration nitrate solutions; their performance in real wastewater remains unclear. Future efforts should focus on developing operando techniques to track *H migration at the nanoscale, testing catalysts under low-concentration and real wastewater conditions, and designing more efficient heterointerfaces to lower the energy barrier for *H transfer.

#### 3.3.2. Reverse Hydrogen Spillover

Classical hydrogen spillover directs *H from strong *H-adsorbers (e.g., Pt, Pd, or water-dissociation sites) to weaker *H-adsorbers or supports. While effective for *H supply, this directional constraint limits the scenario where the final hydrogenation site is itself a strong *H adsorber, a common situation for many transition metals. The concept of reverse hydrogen spillover offers a complementary solution. Different from classical spillover, reverse hydrogen spillover refers to *H migrating from a weak or moderate *H-adsorber (e.g., a nitride, oxide, or even liquid metal) to a stronger metal site, thereby activating the latter for hydrogenation while the former serves as the *H generator. This directionality enables a single active center to undertake both reactant adsorption and hydrogen acceptance, overcoming a key limitation of the classical mechanism [66,67,101,102,103]. Typical examples of reverse hydrogen spillover include the Ni_3_N-Cu system [66], Co@Ga liquid metal system [67], as well as Ru-CoFe LDH@Cu_x_O nanowires [101] and Ru/WO_3-x_ in acidic media [102]. A common feature of these systems is the presence of bridge species (e.g., nitrogen or oxygen) that facilitate directional *H transport.

**Figure 8:**
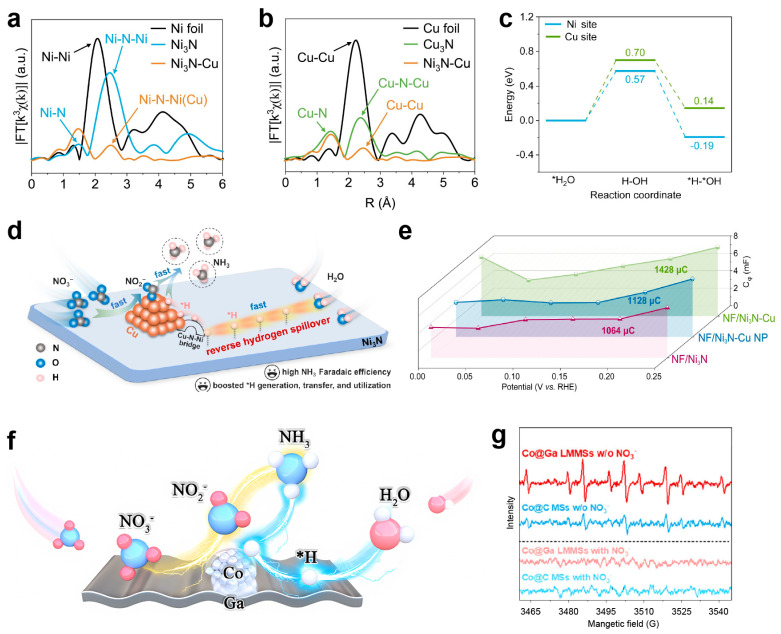
(**a**) FT-EXAFS spectra of Ni foil, Ni_3_N, and Ni_3_N–Cu. (**b**) FT-EXAFS spectra of Cu foil, Cu_3_N, and Ni_3_N–Cu. (**c**) Energy barrier for water dissociation of the Ni_3_N–Cu. (**d**) Schematic illustrations of water dissociation and NO_3_RR on Ni_3_N–Cu with interfacial reverse hydrogen spillover. (**e**) Q_H_ of NF/Ni_3_N–Cu, NF/Ni_3_N–Cu NP, and NF/Ni_3_N. Reprinted with permission from ref. [66], Copyright 2025, WILEY-VCH GmbH. (**f**) Schematic illustration of water dissociation and NO_3_RR on Co@Ga LMMSs. (**g**) Electrochemical quasi in situ EPR tests at −0.3 V versus RHE. Reprinted with permission from ref. [67], Copyright 2025, WILEY-VCH GmbH.

Among these representative systems, the Ni_3_N-Cu architecture stands out as a particularly elegant implementation, where the roles of *H producer, transport bridge, and hydrogenation acceptor are precisely defined and experimentally validated. Ouyang et al. creatively applied the reverse hydrogen spillover mechanism to the NO_3_RR process by constructing a Ni foam-supported Ni_3_N nanosheet catalyst decorated with Cu nanoclusters (NF/Ni_3_N-Cu) [66]. This catalyst achieves 98.7% Faradaic efficiency and a 1.19 mmol h^−1^ cm^−2^ NH_3_ yield at −0.3 V vs. RHE, outperforming most reported catalysts. XAFS in Figure 8a,b confirmed the presence of Ni–N–Cu bridge bonds, providing the structural basis for directional *H transport. A three-site cooperative mechanism was identified as playing a crucial role in this system (Figure 8c,d). Ni sites serve as *H producers due to their low water dissociation energy barrier (0.57 eV). Directional *H migration is then facilitated by the N sites, which act as transport bridges via Ni–N–Cu bonds. Ultimately, the Cu sites take on the dual tasks of adsorbing NO_3_^−^ and delivering *H for hydrogenation. Electrochemical kinetic analysis demonstrated that the introduction of Cu significantly increases the *H adsorption capacity on the Ni_3_N surface, with hydrogen adsorption charge (Q_H_) increasing from 1064 to 1428 μC (Figure 8e), and accelerates *H desorption kinetics. This reverse spillover mechanism enables efficient *H delivery from evolution sites to hydrogenation-active centers, overcoming the limitation of acceptor sites being non-participatory in reactions inherent to classical spillover.

Unlike conventional reverse spillover designs based on solid-state interfaces, Chen et al. exploited the dynamic interfacial properties of liquid gallium to intensify the reverse spillover effect in Co@Ga liquid metal microsphere catalysts (Co@Ga LMMSs) [67]. This system features a unique liquid–solid–electrolyte (L–S–L) dynamic interface, with liquid Ga microspheres as the core and Co nanosheets as the shell. Theoretical calculations reveal that liquid Ga possesses excellent water dissociation capability, exhibiting a Volmer step energy barrier of 0.70 eV that is significantly lower than that of Co sites. Meantime, its relatively high HER barrier positions liquid Ga as an ideal *H producer (Figure 8f). Co sites, in contrast, function as *H consumers responsible for NO_3_^−^ adsorption and hydrogenation (Figure 8f). More remarkably, the fluidity of liquid Ga endows it with a *H diffusion coefficient nearly three times higher than that of solid Ga, substantially accelerating the reverse hydrogen spillover process. In situ EPR spectroscopy (Figure 8g) confirmed *H generation and consumption, while distribution of relaxation time analysis revealed marked acceleration of proton transfer processes. Based on this dynamic interface design, Co@Ga achieves an exceptional 51 mol h^−1^ g_Co_^−1^ NH_3_ yield and outstanding stability over 400 h. In membrane electrode assembly tests, it operates stably even at an industrial-level current density of 1 A cm^−2^.

The above examples demonstrate that both classical and reverse hydrogen spillover provide effective strategies to address the *H supply bottleneck by spatially decoupling *H generation from consumption. Classical spillover directs *H from strong adsorbers to weaker ones or supports, whereas reverse spillover enables migration toward stronger metal sites for hydrogenation. These two directional variants are complementary, and the choice between them depends on the relative *H adsorption strengths of the donor and acceptor sites. A common requirement across both mechanisms is an intimate heterointerface that facilitates directional *H transport, often mediated by bridge species such as nitrogen or oxygen atoms. Despite their promise, several unresolved issues remain, including the lack of direct nanoscale evidence for *H migration, limited performance validation under real wastewater conditions, and the need for heterointerface design to lower the *H transfer barrier. Future efforts should prioritize operando techniques capable of tracking *H migration, testing under low-concentration and real wastewater conditions, and engineering more efficient heterointerfaces. To provide a critical comparison of the three major strategies discussed above, namely BIEFs, atomic-scale active sites, and hydrogen spillover engineering, Table 2 summarizes their core electronic effects, primary functions, key design parameters, advantages, limitations, typical systems, and representative performance metrics. This comparative overview is intended to guide researchers in selecting the most appropriate strategy for specific NO_3_RR applications and to highlight the complementary nature of these approaches.

## 4. In Situ/Operando Characterization for Interfacial Dynamics

In previous sections, we have demonstrated that rational interfacial electronic engineering enables precise manipulation of electronic structures at well-designed material interfaces, including heterointerfaces, single-atom coordination interfaces, phase boundaries, and atomic-scale interfaces. However, these rationally constructed interfaces are not static under realistic reaction conditions. Driven by applied potentials, reactant adsorption, and intermediate conversion, interfacial structure, valence state, coordination environment, and charge distribution undergo continuous dynamic evolution. Such evolution directly determines whether engineered interfaces can maintain anticipated electronic effects, thus fundamentally governing intrinsic activity, selectivity, and stability of electrocatalysts. Therefore, advanced in situ and operando characterization techniques are indispensable for tracking dynamic evolution of these designed interfaces during catalysis [104,105,106,107,108]. These approaches provide direct experimental insights into dynamic restructuring of engineered interfaces, clarify evolution of interfacial electronic effects under working conditions, and ultimately establish a self-consistent relationship between interface design, dynamic behavior, and catalytic performance. This chapter focuses on three representative categories of engineered interfaces and illustrates how in situ spectroscopy, in situ electrochemistry, and in situ imaging reveal their dynamic properties.

### 4.1. In Situ Spectroscopy Tracking Valence and Coordination Dynamics

Conventional ex situ characterization only provides static structural information before and after reactions, which can hardly reflect real electronic states of engineered interfaces under working conditions. In situ X-ray absorption spectroscopy (XAS), in situ X-ray photoelectron spectroscopy (XPS), and in situ Raman spectroscopy enable real-time tracking of valence conversion, coordination variation, charge redistribution, and adsorbate evolution at heterointerfaces and single-atom interfaces during NO_3_RR, offering atomic-level insights into the dynamic origin of interfacial electronic effects [107,109,110,111,112,113]. The dynamic reconstruction of Cu-based oxide–metal interfaces has been quantitatively elucidated using operando spectroscopic tools. For the Cu_2_O–Cu interface engineered for the NO_3_RR, Bai et al. employed operando XAS combined with quasi-in situ XPS and in situ Raman to track potential-driven interfacial evolution [107]. At a low overpotential (0.10 V vs. RHE), Cu(I) species dominate the interface and catalyze two-electron reduction of NO_3_^−^ to NO_2_^−^. As the potential shifts negatively to −0.30 V vs. RHE, Cu(I) is rapidly reduced to metallic Cu(0), whose proportion exceeds 70% within 15 min. Quantitative EXAFS analysis reveals that the Cu–Cu coordination number increases from approximately 4 (at 0.1 V) to about 10 after 30 min at −0.30 V, accompanied by a dramatic decrease in Cu–O signals, providing direct evidence for severe interfacial reconstruction and enhanced electrical conductivity. In situ Raman spectra simultaneously identify characteristic signals of *NO_3_^−^, *NO_2_^−^, and *NH_2_OH intermediates, whose variations are highly consistent with the evolution of Cu valence states, offering direct spectral evidence for understanding how the designed interfaces switch product selectivity through valence regulation.

Single-atom catalysts exhibit distinctive interfacial evolution characterized by dynamical coordination restructuring rather than bulk valence conversion. Using in situ XAFS, Cheng et al. directly observed structural evolution of Fe–N coordination interfaces during NO_3_RR [110]. The initial pyrrole–N_4_–Fe interface undergoes Fe–N bond cleavage under reaction potentials, transforming into a low-coordination pyrrole–N_3_–Fe interface. Such atomic-level interfacial reconstruction effectively tailors the electronic structure and coordination environment of Fe sites, strengthens adsorption and activation of *NO intermediates, and significantly lowers the energy barrier of the *NO → *NHO rate-determining step. This work clarifies the intrinsic mechanism of high activity and selectivity in single-atom catalysts from the perspective of dynamically coordinated electronic effects at atomic interfaces.

Evolution of charge redistribution at heterogeneous interfaces can be effectively captured by combined operando spectroscopic techniques. Geng et al. dynamically monitored laser-programmed Co–Ag dual heterointerfaces using in situ XPS and in situ Raman spectroscopy [111]. In situ XPS measurements revealed that high-valence cobalt species were progressively reduced and eventually converted into metallic Co^0^ during electrochemical activation, forming stable Co–Ag metallic interfaces. Concurrently, in situ Raman spectroscopy tracked sequential evolution of reaction intermediates on the Ag/Co array surface, with characteristic signals corresponding to NO_3_^−^ (1044 cm^−1^), NO_2_^−^ (1332 cm^−1^), and NH_3_-related species (1120 and 1516 cm^−1^), confirming efficient reaction progression along the heterointerfaces. Furthermore, DFT calculations quantified a net interfacial charge transfer of 1.74 e^−^ from Co to Ag, establishing a synergistic electronic configuration with electron-deficient Co sites and electron-rich Ag sites. This unique electronic structure enhances nitrate adsorption and activation while effectively suppressing competing HER. While the above examples focus on tracking dynamic changes, a recent study goes a step further by identifying the origin of reconstruction using operando XAS. Shi et al. employed operando XAS and in situ Raman to investigate Co-based catalysts during NO3RR [114]. Pure Co showed large Co valence fluctuations (0.50 eV) and Co(OH)_2_ formation, whereas the Co_6_Ni_4_ heterostructure remained stable with only 0.06 eV variation. The Ni domains act as an electron reservoir, preventing Co oxidation by compensating the electron deficit caused by adsorbed NO_3_^−^. This work directly demonstrates that operando spectroscopy can identify the fundamental cause of reconstruction, namely an electron supply–demand mismatch, providing a framework for designing dynamically stable interfaces.

### 4.2. In Situ Electrochemistry Probing Kinetics and Intermediate Adsorption

In situ electrochemical methods offer kinetic-level insights into the critical roles of engineered interfaces in regulating reaction rates, pathway branching, and intermediate adsorption behaviors. Serving as an essential complement to in situ spectroscopic characterization, these techniques provide key kinetic evidence for understanding interfacial electronic effects. It should be noted that the term “in situ electrochemistry” in this context specifically refers to hyphenated techniques that couple electrochemical control with mass spectrometry (e.g., DEMS, UME-MS) or impedance spectroscopy (EIS), which provide kinetic and mechanistic information beyond conventional electrochemical measurements.

Identifying short-lived reaction intermediates at atomic coordination interfaces is crucial for unraveling reaction pathways at the molecular level. Zhao et al. developed an ultramicroelectrode–mass spectrometry platform (UME-MS) to perform in situ characterization of atomic-scale coordination interfaces in cobalt-based macrocyclic molecular catalysts [39]. A series of short-lived nitrogen-containing intermediates were successfully detected, including [LCo-NO_3_H]^+^, [LCo-NO_2_]^+^, [LCo-NO_2_H]^+^, [LCo-NO]^+^, [LCo-NHOH]^+^, and [LCo-NH]^+^. Combined with ^15^N isotopic labeling, all nitrogen sources were confirmed to originate from nitrate reduction. By systematically regulating potential and pH value, this study reveals the regulation rules of these parameters on intermediate distribution. Results demonstrate that the electronic structure of the coordination interface directly determines electron/proton transfer rate and reaction pathway selection.

In situ differential electrochemical mass spectrometry (DEMS) enables precise identification of reaction pathways at heterointerfaces. Geng et al. employed in situ DEMS to investigate the NO_3_RR mechanism at Co–Ag heterointerfaces [111]. Characteristic signals at *m*/*z* = 30 (*NO) and *m*/*z* = 31 (*NOH) were clearly observed, confirming *NO as a key gaseous intermediate, while no signal corresponding to *m*/*z* = 33 (NH_2_OH) was detected. Combined with in situ spectroscopic evidence, these results exclude the hydroxylamine pathway and support a sequential hydrogenation pathway that proceeds via *NO → *NOH → *N → *NH → *NH_2_ → NH_3_. Collectively, these findings demonstrate that electronic effects at heterointerfaces directly govern reaction pathway branching, providing crucial kinetic support for mechanistic studies in interface engineering.

### 4.3. In Situ Imaging Visualizing Interfacial Restructuring

In situ electrochemical scanning probe microscopy (EC-AFM/SECM) provides simultaneous nanoscale imaging of both topographic evolution and local electrochemical activity at designed heterogeneous interfaces, offering direct visualization of structural evolution, defect proliferation, and interfacial reconstruction [104,115,116,117]. By integrating experimental observations with theoretical calculations, a comprehensive mechanistic understanding can be established. Dynamic restructuring of amorphous SnO_2_ has been directly visualized using an in situ EC-AFM-SECM platform. Zhao et al. tracked the morphological and activity evolution of this catalyst during NO_3_RR and found that cathodic polarization triggers significant surface roughening, thickness reduction, and defect proliferation [104]. Edges, wrinkles, and defect-rich regions act as active hotspots, showing nearly 100-fold higher activity than flat basal planes. The reconstructed structure, featuring surface Sn^2+^ active sites and subsurface Sn^0^ conductive networks, modulates the electronic structure of Sn sites and lowers the *NO → *NOH rate-determining barrier from 0.85 eV to 0.44 eV. Using nanoscale operando imaging, this work validates that potential-driven interfacial restructuring reshapes electronic effects and spatial activity distribution, establishing a visualized paradigm for understanding the structure–electron–activity relationship in complex electrocatalytic systems.

The in situ and operando techniques discussed above have fundamentally transformed our understanding of NO_3_RR by revealing that catalytically active interfaces are not static but rather dynamically evolve under operating conditions. Valence state transitions, coordination environment rearrangements, and morphological restructuring are not mere side effects but often integral to the emergence of true active sites. As summarized in Table 3, these dynamic behaviors have been captured across various catalyst systems, where operando XAS and in situ Raman track valence state evolution and intermediate identification, DEMS and UME-MS offer kinetic insights into reaction pathways and short-lived intermediate capture, and EC-AFM/SECM visualizes morphological restructuring at the nanoscale. Together, these techniques, facilitated by the comparative overview in Table 3, establish critical links between interfacial electronic effects and catalytic performance.

## 5. Challenges and Prospects

Despite significant progress in interfacial electron engineering for NO_3_RR, several fundamental challenges remain. A primary challenge concerns the delicate balance between *H supply and HER suppression. While strategies such as BIEFs and hydrogen spillover have improved *H management, decoupling *H generation from *H consumption without promoting parasitic H_2_ evolution remains difficult. This requires catalysts with *H adsorption energies precisely tuned near the Sabatier optimum for hydrogenation but not for recombination. Another critical issue is dynamic restructuring of engineered interfaces under operating potentials, including valence changes, coordination evolution, and morphological reconstruction, which undermines predictive design, as the pre-characterized as-prepared interface may not represent the true active state. This calls for in situ/operando techniques with higher temporal and spatial resolution to capture transient active configurations. In addition, despite advances in dual-atom and asymmetric coordination designs, linear scaling relations still couple the adsorption energies of different intermediates (NO_3_^−^, *NO, *H, *NH_2_OH), restricting independent optimization of each step. Overcoming these scaling constraints may require dynamic or multi-site cooperative mechanisms. Addressing these challenges will require concerted efforts across multiple frontiers. Looking ahead, several promising directions are poised to drive the next wave of innovation in interfacial electron engineering for NO_3_RR.

(1) High-throughput DFT calculations combined with machine learning offer a powerful route to accelerate catalyst discovery by screening thousands of heterojunctions, coordination environments, and spillover interfaces. However, for NO_3_RR, the screening criteria must go beyond single descriptors such as the d-band center or nitrate adsorption energy alone. Given the multi-step nature of NO_3_RR (involving eight electrons and nine protons), machine learning models should be trained to simultaneously optimize the adsorption energies of multiple key intermediates, such as *NO_3_, *NO, and *H, to avoid the tradeoffs that arise from linear scaling relations. The optimal design space for BIEF systems, for instance, would involve identifying pairs of materials with a work-function difference (ΔΦ) that simultaneously promotes nitrate adsorption on electron-deficient sites and water dissociation on electron-rich sites. For single-atom and dual-atom catalysts, machine learning can help map the relationship between coordination environment, d-band center, and the binding strength of *NO intermediates, thereby guiding the selection of metal–ligand combinations that break scaling constraints. Experimental validation of computationally predicted unconventional interfaces is expected to accelerate the discovery cycle.

(2) Instead of pursuing static, rigid interfaces, future designs may embrace dynamic adaptability, e.g., using stimuli-responsive materials (with reversible phase transitions or reversible oxygen vacancy formation) that adapt their electronic structure to reaction potential or local microenvironment. In particular, reversible oxygen vacancy formation can serve as a dynamic electronic switch that temporarily modifies the d-band center and charge distribution of active sites. This modulation enables potential-dependent optimization of nitrate adsorption, *H supply, and intermediate desorption, a functionality that static interfaces cannot offer. Once the cathodic potential is removed, these vacancies can be healed, and the original electronic configuration is restored, allowing the catalyst to self-regulate across different stages of the multi-step NO_3_RR cascade.

(3) Next-generation in situ techniques (e.g., operando electrochemical mass spectrometry coupled with microfluidic cells) should be adapted to realistic MEA configurations. For NO_3_RR, three specific dynamic processes deserve particular attention. First, operando XAS with sub-second time resolution is needed to track the transient evolution of BIEFs under pulsed potential operation, as the charge redistribution across heterointerfaces may respond to potential changes on a much faster timescale than currently appreciated. Second, in situ Raman and infrared spectroscopy should be pushed to lower wavenumber regions to capture the vibrational signatures of *NOH versus *NHO intermediates, thereby clarifying the branching point between NH_3_-producing and by-product pathways. Third, operando scanning probe techniques (e.g., EC-AFM/SECM) with improved spatial resolution could provide the first direct visualization of *H migration across heterointerfaces, addressing a long-standing gap in hydrogen spillover research. Correlating these dynamic interfacial changes with time-resolved product evolution will close the loop between design, characterization, and performance.

(4) Bridging the gap between electronic descriptors and catalytic performance: Despite widespread use of d-band center and charge transfer as design descriptors, a quantitative correlation between these electronic parameters and the reaction barriers of individual NO_3_RR steps remains elusive under operando conditions. For NO_3_RR, the challenge is particularly acute because the optimal descriptor for nitrate activation (e.g., charge transfer to *NO_3_ antibonding orbitals) may differ from that for *H supply (e.g., *H adsorption energy) and for intermediate desorption (e.g., N–O binding strength). A single descriptor is unlikely to capture all three requirements. Future efforts should integrate in situ spectroscopy with theoretical calculations to establish dynamic descriptor-activity relationships, enabling predictive design of interfacial electronic structures that adapt to each step of the reaction cascade.

(5) Synergistic integration of multiple electronic effects within a single interface: Current strategies, including BIEFs, single-atom coordination, and hydrogen spillover, are typically developed independently. However, the most efficient NO_3_RR catalysis likely requires their synergistic combination within a single hierarchical architecture. The rationale is rooted in the three kinetic bottlenecks of NO_3_RR. BIEFs could drive charge separation to optimize both nitrate adsorption on electron-deficient sites and water dissociation on electron-rich sites. Single-atom or dual-atom sites could be deliberately placed at the electron-rich regions to achieve precise tuning of intermediate binding energies and break scaling relations. Hydrogen spillover pathways could then be engineered to channel the *H generated at the electron-rich sites toward the atomic-scale hydrogenation centers, ensuring efficient proton delivery without promoting parasitic HER. This three-component integration, namely charge redistribution (BIEF), binding-energy optimization (atomic sites), and proton delivery (spillover), addresses the three kinetic bottlenecks in a single architecture. The key challenge lies in the spatial arrangement: the distance between the *H generation zone and the hydrogenation sites must be carefully controlled to minimize *H recombination before it reaches the intended target. Advanced nanofabrication techniques, such as layer-by-layer assembly or site-selective deposition, may offer pathways to realize such hierarchical interfaces.

## Figures and Tables

**Table 1:** Summary of representative electrocatalysts for NO_3_RR via interfacial electron engineering and their catalytic performance.

Catalyst	Electrolyte	NH_3_ Yield	FE	Ref.
CuO/NiO	0.05 mol·L^−1^ Na_2_SO_4_ + 100 mg·L^−1^ NO_3_^−^	8.87 mg NH_4_^+^-N cm^−2^·h^−1^	61.0%	[52]
Co_3_O_4_/CeO_2_@NCNTs	0.1 M Na_2_SO_4_ + 1.0 M NaNO_3_	14.85 mg·h^−1^·cm^−2^	97.1%	[53]
CoO/Cu	0.4 mol·L^−1^ Na_2_SO_4_ + 0.04 mol·L^−1^ NO_3_^−^	4.3 mg·cm^−2^·h^−1^	96.7%	[54]
c-Co_3_O_4_/a-CuO	0.2 M K_2_SO_4_ + 0.1 M KNO_3_	412.5 μmol·h^−1^·mg^−1^	90%	[55]
Bi_2_S_3_-Bi_2_O_3_	0.1 M KHCO_3_ + 50 mM KNO_3_	89.83 mg·g^−1^·h^−1^	94%	[56]
Co(OH)_2_/CoO	1 M KOH + 0.1 M KNO_3_	73.9 mg·h^−1^·cm^−2^	95.6%	[57]
Cu_x_S-Co_0_._5_	1 M KOH + 0.1 M KNO_3_	5.36 mg h^−1^ cm^−2^	95.6%	[58]
TA-Cu^2+^-CuO	0.5 M K_2_SO_4_ + 2000 ppm KNO_3_	0.99 mmol·h^−1^·cm^−2^	99.4%	[59]
PR-CuNC	0.1 M KOH + 0.1 M KNO_3_	3.74 mg·h^−1^·cm^−2^	94.6%	[60]
Cu–Fe DAC/NC	1 M KOH + 0.1 M KNO_3_	6.0 mg·cm^−2^·h^−1^	94.3%	[61]
Fe-Mn/SNC	1 M NaOH + 0.1 M NaNO_3_	57.4 μmol·h^−1^·cm^−2^	98.7%	[62]
RuCu DAs/NGA	0.1 M KOH + 0.1 M KNO_3_	3.1 mg·h^−1^·cm^−2^	95.7%	[63]
Ni_2_P/Pd_6_P	0.5 M Na_2_SO_4_ + 0.05 M NO_3_^−^	0.908 mmol·h^−1^·mg^−1^	92.6%	[64]
CoNi–LDH@Cu_2_O	1 M NaOH + 0.1 M NaNO_3_	75.2 mg·h^−1^·cm^−2^	97.8%	[65]
NF/Ni_3_N-Cu	1 M KOH + 0.1 M KNO_3_	1.19 mmol·h^−1^·cm^−2^	98.7%	[66]
Co@Ga LMMSs	1.0 M NaOH + 1.0 M NaNO_3_	51 mol·h^−1^·g^−1^	94.5%	[67]

**Table 2:** Comparison of the three major interfacial electron engineering strategies for NO_3_RR: built-in electric fields (BIEFs), atomic-scale active sites, and hydrogen spillover engineering.

Aspect	BIEFs	Atomic-Scale Sites	Hydrogen/Reverse Hydrogen Spillover
Core electronic effect	Charge redistribution across heterointerface	d-band center modulation and d–p orbital coupling	Spatial separation of *H generation and consumption
Primary function	Simultaneous enhancement of NO_3_^−^ adsorption, *H supply, and intermediate tuning	Precise optimization of intermediate adsorption energies	Decoupling *H production from hydrogenation sites
Key parameter	Work function difference (ΔΦ)	Coordination number, heteroatom type	*H adsorption energy difference (ΔG)
Advantage	Multifunctional synergy within a single interface	Breaking linear scaling relations; high atom utilization	Suppressing HER while ensuring *H supply for hydrogenation
Limitation	Interface stability; unclear BIEF evolution	Low site density; structural degradation and aggregation under reaction conditions	Indirect evidence of *H migration; limited testing in real wastewater

**Table 3:** Summary of in situ/operando characterization techniques applied to representative catalyst systems for NO_3_RR.

Catalyst System	In Situ/Operando Technique	Key Dynamic Information Revealed	Ref.
Cu_2_O nanocubes	Operando XAS, in situ Raman	Cu(I) → Cu(0) reduction; intermediate evolution (*NO_3_, *NO_2_, *NH_2_OH)	[107]
Fe-N-C single atom	In situ XAFS	Structural reconstruction from pyrrole-N_4_–Fe to pyrrole-N_3_–Fe	[110]
Co-Ag dual heterojunction	In situ XPS, in situ Raman, DEMS	Valence state evolution, intermediate identification, reaction pathway (*NO → *NOH → *N → *NH → *NH_2_ → NH_3_)	[111]
Co6Ni4 heterostructure	Operando XAS, in situ Raman	Co valence stabilization, Ni domains as electron reservoir preventing Co oxidation	[114]
Amorphous SnO_2_	EC-AFM-SECM	Surface roughening, defect proliferation, active hot spot distribution	[104]
Co-based macrocyclic molecular catalyst	UME-MS	Short-lived intermediate capture (*NO, *NHOH, etc.)	[39]

## Data Availability

No new data were created or analyzed in this study.
